# Responsible Use of Large Language Models in Microbial Genomics and Bioinformatics: A Life-Science Framework for Reliability, Reproducibility, and Risk-Aware Interpretation

**DOI:** 10.3390/life16061032

**Published:** 2026-06-20

**Authors:** Mia Yang Ang, Li Chen, Lanni Song, Leonard Lipovich, Siew Woh Choo

**Affiliations:** 1 Department of Biomedical Sciences, Jeffrey Cheah Sunway Medical School, Faculty of Medical and Life Sciences, Sunway University, Sunway City, Petaling Jaya 47500, Selangor, Malaysia; 2 Sunway Microbiome Centre, Faculty of Medical and Life Sciences, Sunway University, Sunway City, Petaling Jaya 47500, Selangor, Malaysia; 3 Zhejiang-Malaysia Joint Laboratory for Rare Medicinal Resources, Wenzhou-Kean University, 88 Daxue Road, Ouhai, Wenzhou 325060, China; 4 College of Science, Mathematics and Technology, Wenzhou-Kean University, 88 Daxue Road, Ouhai, Wenzhou 325060, China; 5 Dorothy and George Hennings College of Science, Mathematics and Technology, Kean University, 1000 Morris Ave, Union, NJ 07083, USA; 6 International Frontier Interdisciplinary Research Institute (IFIRI), Wenzhou-Kean University, 88 Daxue Road, Ouhai, Wenzhou 325060, China

**Keywords:** large language models, microbial genomics, bioinformatics, antimicrobial resistance, metagenomics, microbiome, reproducibility, benchmarking, responsible AI, computational biology

## Abstract

Large language models (LLMs) are increasingly adopted in life-science research for scientific writing, coding, literature synthesis, workflow troubleshooting, and preliminary data interpretation. In microbial genomics and bioinformatics, their appeal is clear because researchers routinely integrate genome annotations, antimicrobial resistance profiles, virulence determinants, taxonomic assignments, microbiome outputs, workflow scripts, and primary literature. Yet this domain also highlights major risks, including hallucinated biological claims, inaccurate citations, irreproducible code, unsupported genotype-to-phenotype inference, and inappropriate clinical or public health framing. This narrative review examines responsible LLM use in microbial genomics as a representative life-science setting where interpretation depends on database provenance, validated workflows, expert assessment, and reproducible evidence chains. It considers applications in genome annotation, antimicrobial resistance interpretation, virulence analysis, microbiome and metagenomics workflows, coding support, and scientific writing. The review further presents MicrobeGuardGPT as a conceptual reliability framework for assessing LLM-assisted microbial genomics outputs before scientific, clinical, or public health use. By connecting task domains, evidence verification, expert validation, and reliability classification, the framework supports risk-aware LLM integration in bioinformatics. Responsible implementation will require domain-specific benchmarks, curated database linkage, transparent reporting, reproducible workflows, human oversight, and governance standards tailored to biological interpretation across research, diagnostic, surveillance, outbreak-response, educational, and translational contexts.

## 1. Introduction

Large language models (LLMs) are increasingly being explored as assistive tools in biomedical research, bioinformatics, and scientific writing [1]. Their use in explanation, summarization, question answering, coding support, and scientific text drafting reflects the rapid expansion of transformer-based and LLM applications in healthcare and biomedical research [2]. In microbial genomics, where researchers often work with genome annotations, antimicrobial resistance (AMR) profiles, metagenomic tables, pathogen surveillance reports, workflow scripts, and large bodies of literature, LLMs appear attractive as tools for interpretation support and scientific communication.

### 1.1. Opportunities and Risks of LLM Use in Microbial Genomics

Microbial genomics is a challenging context for LLM-assisted interpretation. It requires reasoning across genes, genomes, strains, species, mobile genetic elements, phenotypes, ecological context, metadata, and experimental design. Genome annotation commonly depends on automated bacterial annotation systems, curated functional resources, and tool-specific rules [3]. For example, detecting an AMR determinant is not equivalent to confirming a resistance phenotype, because interpretation depends on database thresholds, gene identity, sequence quality, organism background, genomic context, and phenotypic validation [4]. Similarly, the presence of a virulence-associated gene does not automatically establish pathogenic potential or clinical risk [5]. These distinctions are central to microbiology but may not be handled reliably by general-purpose LLMs without domain-specific verification.

LLMs may assist with annotation interpretation, AMR explanation, microbiome result summarization, workflow troubleshooting, code generation, literature synthesis, and manuscript preparation. However, without verification, their outputs may contain inaccurate, unsupported, irreproducible, or misleading biological and technical statements [6].

### 1.2. Need for Reliability, Reproducibility, and Domain-Specific Verification

Existing biomedical LLM discussions provide useful guidance on general opportunities, risks, reporting needs, and responsible AI use, but they do not fully address microbial-genomics-specific reliability challenges [1]. Reliability in this field depends not only on factual accuracy but also on biological context, database versioning, workflow validity, uncertainty handling, and expert microbiological review. Reproducibility and data stewardship principles are therefore essential for determining whether LLM-assisted outputs can be made transparent, auditable, and scientifically accountable [7].

Microbial genomics provides a useful model domain for examining responsible LLM use in the life sciences because it combines biological complexity, computational workflows, database-dependent interpretation, and potential clinical or public health relevance. Errors in this setting may arise not only from incorrect language generation, but also from weak linkage between genomic evidence, organism context, analytical parameters, database versions, and downstream interpretation. Therefore, lessons from microbial genomics are relevant to broader life-science bioinformatics, where AI-assisted reasoning must remain reproducible, evidence-supported, and expert-validated.

### 1.3. Scope and Contribution of This Review

Because this article proposes a conceptual framework and research agenda rather than an empirical benchmark study, it is positioned as a narrative review and theoretical contribution for life-science bioinformatics. The goal is to define evaluation dimensions and reporting safeguards that can guide future benchmark development, software implementation, database-linked validation, and expert-review workflows.

This narrative review and conceptual framework article critically examines the role of LLMs in microbial genomics and life-science bioinformatics, with emphasis on genome annotation, antimicrobial resistance interpretation, virulence and pathogenicity reasoning, microbiome and metagenomics interpretation, workflow support, and scientific writing. To address these gaps, the review introduces MicrobeGuardGPT as a conceptual reliability framework for evaluating LLM-assisted microbial genomics outputs. The review argues that responsible integration of LLMs into life-science bioinformatics requires domain-specific benchmark development, curated database linkage, transparent reporting, reproducible workflows, and human expert review [8].

## 2. Foundations of Large Language Models for Bioinformatics

Large language models are artificial intelligence systems designed to process and generate text based on statistical patterns learned from large training datasets [9]. Contemporary healthcare and biomedical LLMs build on transformer-based language modeling and large-scale pretraining, enabling tasks such as explanation, summarization, question answering, code generation, and scientific text drafting [2]. In bioinformatics, their appeal reflects the field’s mixed linguistic and computational workload, where researchers frequently move between biological concepts, command-line tools, databases, scripts, metadata files, annotation tables, and manuscript text.

### 2.1. General-Purpose LLMs and Scientific Reasoning

General-purpose LLM systems can perform broad language- and code-related tasks [10]. Their flexibility is useful across scientific settings, especially where researchers need to explain concepts, summarize text, generate scripts, or organize technical information. In microbial genomics, this flexibility is attractive because interpretation often requires both biological and computational reasoning.

However, general-purpose capability does not necessarily indicate domain-specific reliability [8]. LLMs are not replacements for genome assemblers, taxonomic classifiers, antimicrobial resistance prediction pipelines, variant callers, statistical software, or workflow engines. A model may correctly describe a broad concept but still misapply it to a specific genome, taxon, AMR profile, or metagenomic dataset. Their role in bioinformatics should therefore remain assistive and subject to domain-specific verification [11].

### 2.2. Biomedical LLMs and Biological Foundation Models

Biomedical LLMs are adapted using biomedical literature, clinical text, or domain-specific corpora, which may make them more familiar with scientific terminology and biomedical writing conventions [12]. Medical LLM evaluations suggest that domain-specific benchmarking is needed before such models are used in high-stakes biomedical contexts [8]. However, biomedical language familiarity is not equivalent to biological validation, and these models may still hallucinate references, misinterpret evidence, or overstate findings [13].

Biological foundation models differ because they learn directly from biological sequences such as DNA, RNA, or proteins [14]. These models may support sequence-function prediction, protein representation, variant effect prediction, or structure-related tasks. Protein language models illustrate how sequence-scale learning can support structural or functional inference [15]. Although biological foundation models are sometimes discussed alongside conversational LLMs, their inputs, outputs, and validation needs differ substantially from text-generating systems used for explanation or writing.

For reliability assessment, it is useful to distinguish four partially overlapping system types. General text-based LLMs primarily generate natural-language or code outputs from broad pretraining data. Domain-adapted biomedical or scientific LLMs may better recognize biomedical terminology and writing conventions but still require claim-level checking in microbial genomics [12]. Sequence-trained biological foundation models, especially protein language models, learn representations from biological sequences and may support sequence-function or structure-related prediction, but they are not automatically evidence-aware interpreters of genome annotations, AMR profiles, or microbiome results [14,15]. Hybrid systems combine language models with retrieval, databases, workflow engines, or code execution environments; these systems can improve provenance and reproducibility only when the retrieved sources, database versions, parameters, and executed outputs are explicitly documented and reviewed.

These distinctions are important because reliability failures differ by model class. A text-based LLM may provide a fluent but unsupported biological explanation; a sequence model may produce a score that is difficult to interpret biologically; and a retrieval-augmented system may cite a real source while still applying it to the wrong organism, database release, or analytical context. Therefore, domain specialization should be treated as a reason for more targeted validation, not as evidence that validation is no longer required.

### 2.3. Retrieval-Augmented and Tool-Linked LLM Systems

Retrieval-augmented LLM systems connect an LLM to external sources such as scientific literature, curated databases, uploaded documents, or institutional knowledge bases, which may reduce unsupported answers but does not eliminate errors in retrieval quality, source selection, citation accuracy, or interpretation [16]. For microbial genomics, retrieval-augmented systems may be useful when linked to validated resources and accompanied by transparent evidence checking.

Tool-linked LLM systems may also support bioinformatics by interacting with code interpreters, workflow environments, database queries, or document repositories [11]. These systems could help users draft scripts, check outputs, summarize files, or organize reproducible analyses. However, tool access does not automatically guarantee correctness. Outputs still require validation of input files, tool versions, parameters, database versions, code execution, and biological interpretation [17].

These distinctions provide the basis for considering how LLMs may be applied across microbial genomics workflows.

## 3. Applications of LLMs in Microbial Genomics and Bioinformatics

LLMs may have practical value across several stages of microbial genomics and bioinformatics, particularly when computational outputs need to be explained, organized, or translated into scientific language [1]. Their possible applications span diverse input data types, including genome assemblies, annotation files, AMR gene profiles, virulence factor outputs, metagenomic abundance tables, pathway results, workflow scripts, and literature sources [5]. These inputs can support LLM-assisted tasks such as annotation explanation, AMR interpretation, microbiome reasoning, coding support, workflow troubleshooting, and literature synthesis. The resulting outputs may include interpretation reports, workflow scripts, summaries, teaching materials, manuscript sections, and preliminary hypotheses.

**Figure 1:**
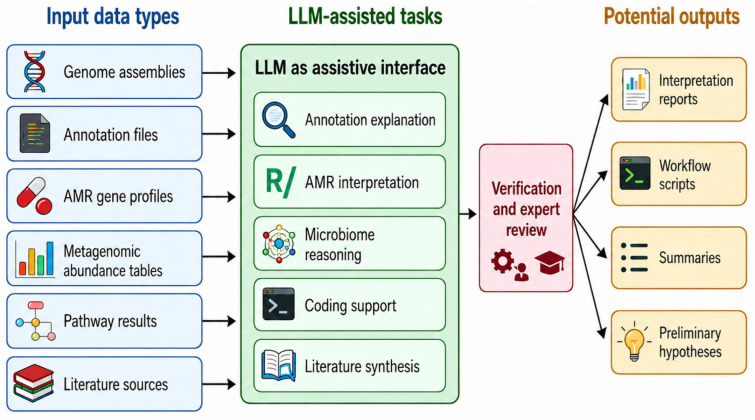
Landscape of LLM applications in microbial genomics. The diagram links microbial genomics inputs, including genome assemblies, annotation tables, AMR profiles, virulence-factor outputs, metagenomic profiles, workflow scripts, and literature sources, to LLM-assisted tasks such as explanation, coding support, troubleshooting, summarization, and hypothesis generation. The validation layer indicates that LLM-assisted outputs must be checked against curated databases, documented software outputs, versioned workflows, primary literature, and relevant metadata. Expert oversight determines whether the final output is suitable for education, drafting, research interpretation, or higher-risk clinical or public-health contexts. The figure is conceptual and does not represent an independently validated decision-support pipeline.

These applications should be understood as assistive rather than definitive [17]. Figure 1 summarizes this assistive role across microbial genomics inputs, LLM-assisted tasks, and potential outputs.

### 3.1. Genome Annotation and Functional Interpretation

Genome annotation is one area in which LLMs may support microbial genomics. Annotation outputs often include predicted genes, protein names, enzyme functions, pathway terms, locus tags, mobile element annotations, and hypothetical proteins [3]. These outputs are essential for interpreting microbial genomes, but they can be difficult to synthesize, especially when users must compare multiple annotation systems or explain findings to interdisciplinary audiences. LLMs may help summarize annotation tables, explain predicted gene functions, draft genome feature descriptions, and translate technical annotation outputs into clearer biological language.

Functional interpretation should remain tied to validated annotation systems and curated resources [3]. Genome and pathway interpretation may draw on KEGG [18], Prokka [19], RASTtk [20], KofamKOALA [21], COG [22], UniProt [23], and RefSeq [24] curation. Gene-calling and annotation quality may also be influenced by tools such as Prodigal [25], Bakta [26], and assembly pipelines such as SPAdes [27]. LLM-assisted annotation summaries should therefore be checked against database evidence, organism context, genome quality, and annotation confidence rather than inferred from gene names alone.

Genome assembly and taxonomic context are also important for interpretation. Assembly quality should remain anchored to established metrics from tools such as QUAST [28], while completeness and contamination can be assessed using tools such as CheckM [29]. Taxonomic placement should be interpreted using documented taxonomic frameworks and tools such as GTDB-Tk [30].

### 3.2. AMR Interpretation and Resistome Reasoning

Antimicrobial resistance interpretation is an important application area because AMR is biologically complex and may have clinical or public health implications [4]. LLMs may help explain resistance gene families, resistance mechanisms, antimicrobial drug classes, and the distinction between genotype and phenotype. They may also assist in drafting cautious result summaries that distinguish detected resistance determinants from experimentally confirmed resistance.

However, AMR interpretation requires strong caution. The detection of an AMR determinant does not by itself confirm phenotypic resistance, clinical treatment failure, or transmission risk [4]. Interpretation depends on gene identity, allelic variation, expression, promoter context, copy number, organism background, plasmid or chromosomal location, mobile genetic element context, genome quality, and antimicrobial susceptibility testing [31]. LLM-assisted AMR explanations should therefore be checked against established AMR resources and documented tool outputs, including CARD [32], ResFinder [31], AMRFinderPlus [33], MEGARes [34], ARG-ANNOT [35], and PointFinder [36].

Additional evidence streams may also be required depending on the research question. Resistance to metals, biocides, and disinfectants may require resources such as BacMet [37], while plasmid-mediated dissemination may require tools such as PlasmidFinder [38]. Whole-genome sequencing can inform antimicrobial susceptibility prediction, but it does not replace phenotypic testing in all settings [4].

### 3.3. Virulence, Pathogenicity, and Risk Interpretation

LLMs may also assist in explaining virulence-associated genes, pathogenicity islands, secretion systems, toxins, adhesins, immune evasion factors, and host-associated traits. Such outputs are common in microbial genome analysis, but they require careful biological interpretation. A model may help summarize known functions of a virulence factor, explain its possible role in host interaction, or distinguish experimentally characterized virulence factors from predicted homologues.

Virulence interpretation should remain grounded in curated resources and organism-specific evidence. Databases such as VFDB [39], Victors [40], and PHI-base [41] can support the interpretation of virulence-related features. IslandViewer [42] may provide genomic island context, while pathogen genome sequencing studies [5] illustrate why genomic context is essential for interpreting virulence-associated loci.

The main risk is biological overinterpretation. The presence of a virulence-associated gene should be interpreted as potential evidence requiring genomic, functional, organism-specific, and, where relevant, epidemiological support [5]. LLM outputs in this area should therefore distinguish between gene presence, predicted function, experimentally validated function, and disease relevance.

### 3.4. Microbiome and Metagenomics Interpretation

Microbiome and metagenomics analysis is another area where LLMs may be useful, particularly for explaining workflows, summarizing outputs, and drafting interpretation. Amplicon workflows such as QIIME 2 [43] and DADA2 [44], together with marker-gene databases such as SILVA [45] and Greengenes [46], are common foundations for microbiome analysis. LLMs may help explain alpha and beta diversity, taxonomic composition, differential abundance outputs, and ecological interpretation, but they should not replace statistical modeling or domain review [47].

Because microbiome taxonomic interpretation is sensitive to database choice and release date, older resources should be described with care. Greengenes [46] remains historically important for many legacy 16S rRNA workflows, and Greengenes2 [48] provides a more recent reference-tree update. Current analyses may instead use updated marker-gene resources such as SILVA [45] or genome-based taxonomic frameworks such as GTDB/GTDB-Tk [30,49], depending on whether the data are amplicon, metagenomic, isolate-genome, or metagenome-assembled-genome data. LLM-assisted interpretation should therefore record the database, release, classifier, confidence threshold, and taxonomic framework used before drawing biological conclusions.

Shotgun metagenomic interpretation may involve taxonomic, functional, and strain-level profiling tools, each with different assumptions, databases, and limitations [50]. Marker-based approaches such as MetaPhlAn [51], broader workflow ecosystems such as bioBakery [50], and functional profiling tools such as HUMAnN [52] support different aspects of metagenomic analysis. PICRUSt2 [53], Bracken [54], and Kaiju [55] address related taxonomic or functional inference tasks, but their assumptions and input requirements differ. Therefore, LLM-assisted explanations of metagenomic or predicted functional outputs should be checked against the software used, database version, taxonomic resolution, statistical model, input data type, and study design.

The risk of overinterpretation is especially high in microbiome studies. An LLM might describe an increase in a bacterial taxon as beneficial, harmful, diagnostic, or causal without considering study design, sequencing depth, batch effects, compositionality, confounding, multiple testing, or statistical uncertainty [56]. Best-practice microbiome analysis and compositional-data principles remain essential for interpreting abundance data [47].

### 3.5. Coding, Workflows, Literature Synthesis, and Scientific Writing

Beyond biological interpretation, coding assistance is one of the most common practical uses of LLMs in bioinformatics [57]. LLMs can generate R, Python, Bash, Perl, Snakemake, or Nextflow scripts; explain error messages; suggest workflow structures; draft README files; and help users understand command-line tools [57,58]. For microbial genomics, this may include scripts for parsing annotation files, filtering AMR gene tables, summarizing taxonomic profiles, merging metadata, preparing figures, or automating routine quality checks.

Generated code may contain limitations that are not evident from syntax alone and should be tested on representative input data [59]. Reproducible workflow systems such as Snakemake [60] and Nextflow [61], package management systems such as Bioconda [62], and container platforms such as Singularity [63] can improve portability when code is curated, tested, and documented. Broader reproducibility and scientific-computing guidance also emphasizes provenance, testing, documentation, and explicit dependencies [17].

LLMs can also assist with literature summarization, manuscript outlining, paragraph drafting, language refinement, and thematic organization. In review writing, they may help identify conceptual gaps, compare study findings, and improve readability. Reporting standards for reviews reinforce that source selection, screening, and synthesis should remain explicit, even when LLMs are used for drafting or organization [64]. LLMs may fabricate references, provide incorrect PMIDs or DOIs, cite real papers for unsupported claims, or summarize findings inaccurately [13]. LLM-assisted writing therefore requires manual reference checking, primary literature review, and expert revision [6].

Because LLM-assisted tasks vary in scientific and public health risk, they should not be evaluated as a single category of use. Table 1 provides a risk-stratified view of common use cases in microbial genomics and bioinformatics, together with acceptable roles and required safeguards.

Methodological note on risk categories: The qualitative risk levels in Table 1 were assigned according to the potential consequence of an incorrect output, the degree of biological inference required, dependence on database currency and provenance, reproducibility requirements, need for expert review, and likelihood of overinterpretation or hallucination. Microbiome interpretation is therefore rated as moderate to high because taxonomic and functional summaries can be strongly affected by study design, compositionality, batch effects, database release, statistical modeling, and causal overstatement, even when the immediate clinical consequence is lower than for AMR or outbreak-related interpretation.

## 4. Risks and Limitations of LLM Use in Microbial Genomics

Although LLMs can support microbial genomics and bioinformatics, their outputs require careful interpretation. These models generate responses from learned language patterns rather than direct biological validation [9]. As a result, they may produce scientifically plausible language that is incorrect, unsupported, incomplete, irreproducible, or unsafe [6]. In microbial genomics, these risks are particularly important because outputs may involve antimicrobial resistance, virulence, pathogen surveillance, microbiome-disease associations, or workflow decisions with clinical or public health relevance [4].

### 4.1. Core Risks Across LLM-Assisted Microbial Genomics

The main risks can be consolidated into four recurring categories. First, factual and citation errors occur when an LLM-assisted output invents genes, mechanisms, tools, references, PMIDs, DOIs, or database claims. Second, biological overinterpretation occurs when gene presence, taxonomic abundance, sequence similarity, or predicted function is presented as phenotype, causality, pathogenicity, or actionability without adequate evidence. Third, provenance and reproducibility failures occur when model version, prompt context, software settings, database release, input data, or validation steps are not recorded. Fourth, safety and governance risks arise when outputs involving AMR, virulence, pathogen surveillance, clinical genomics, or outbreak response are treated as decision support without qualified review.

The following subsections discuss these risks separately, but they should be evaluated together in practice. For example, an AMR interpretation may be factually plausible but still unreliable if it lacks database provenance, ignores organism-specific context, or implies treatment relevance without susceptibility testing and expert review.

### 4.2. Hallucination of Biological and Technical Information

Hallucination is one of the most important risks of LLM use in scientific interpretation [6]. In microbial genomics, hallucination may involve invented gene names, incorrect resistance mechanisms, unsupported pathway descriptions, fabricated software tools, false parameter recommendations, or non-existent references [13]. These errors can be subtle because they may be embedded within otherwise fluent and convincing scientific language.

Hallucinated content is particularly concerning when it affects biological interpretation. Fabricated gene functions, incorrect DOIs, or unsupported resistance mechanisms may be difficult to detect without checking against curated databases, original publications, validated analytical outputs, and software documentation [11].

### 4.3. Biological Inconsistency and Overinterpretation

Biological inconsistency often arises when LLM-assisted interpretation ignores microbial scale and context. An output may conflate genus-, species-, strain-, or lineage-level evidence, transfer observations from one organism to another, or overlook plasmid location, mobile elements, operon structure, promoter context, gene truncation, assembly quality, and sample metadata. For this reason, biological claims should be checked against the organism, sequence evidence, database release, analytical method, and intended use before they are accepted.

This limitation is important in AMR, virulence, and microbiome studies. The presence of a resistance gene does not always indicate phenotypic resistance; detection of a virulence-associated gene does not prove pathogenicity [4]; and microbial abundance differences do not establish causality [56]. These interpretive constraints are well recognized in AMR prediction and microbiome analysis [4,47].

### 4.4. Reproducibility Problems

Reproducibility is central to bioinformatics, yet LLM-assisted outputs may vary with prompt wording, session context, model version, and configuration [17]. The same question may produce different explanations, code, or levels of caution, creating challenges for transparent computational research [8]. This variability is problematic if LLM assistance is not documented.

In coding and workflow assistance, reproducibility problems may appear as inconsistent package choices, changing parameter recommendations, incomplete documentation, or scripts that run in one environment but fail in another. These issues are particularly important in microbial genomics, where reproducibility depends on software versions, database versions, reference genomes, parameter choices, and metadata handling [17]. Another source of poor reproducibility is variable quality in public genome assemblies and annotations. LLM-assisted interpretation may incorrectly treat all public genomic records as equally reliable, even when annotations are based mainly on sequence similarity or open reading frame prediction without transcriptomic, proteomic, or experimental support. This is particularly important for non-model organisms or poorly characterized taxa. LLM-assisted workflows for microbial genomics should therefore include safeguards for annotation quality, evidence level, database provenance, and organism-specific context.

### 4.5. Citation and Evidence Reliability

Citation reliability is a major concern in LLM-assisted scientific writing [13]. LLMs may generate fake references, incorrect PMIDs, inaccurate DOIs, or real papers that do not support the claims being made [13]. They may also summarize a paper inaccurately, exaggerate its conclusions, or transfer findings from one organism, dataset, or clinical context to another.

Authors should verify every LLM-suggested reference manually using PubMed, journal websites, official database documentation, and primary publications [6]. This is especially important in review articles, where citation errors can weaken the credibility of the entire manuscript [59].

Mitigation requires more than confirming that a citation exists. Each LLM-assisted citation should be checked for DOI, PMID, URL, authorship, publication year, and journal details; the cited paper should then be read or inspected to confirm that it actually supports the specific claim. For manuscript preparation, authors should avoid allowing an LLM to insert unsupported references directly, maintain a citation-audit trail, and verify database or software claims against official documentation and release notes.

### 4.6. Code and Workflow Reliability

LLMs can be useful for generating bioinformatics scripts, but code reliability remains a major limitation [57]. Generated scripts may contain syntax errors, outdated functions, missing dependencies, inefficient logic, inappropriate file handling, or incorrect statistical assumptions [10]. Even executable scripts may use unsuitable normalization, incorrect metadata grouping, or inappropriate statistical testing.

A workflow may appear complete but omit quality control, database versioning, logging, provenance tracking, or parameter documentation. Best-practice guidance for scientific computing emphasizes testing, version control, documentation, and dependency management as basic requirements for reliable computational work [59]. Containerization and workflow systems can reduce risk only when they are integrated into the analysis rather than added retrospectively [63].

### 4.7. Unsafe Clinical or Public Health Interpretation

Some microbial genomics outputs may have clinical or public health relevance, especially in AMR surveillance, pathogen genomics, outbreak investigation, and infectious disease research [65]. In these settings, LLMs may generate interpretations that appear clinically meaningful but are not supported by validated pipelines, phenotypic testing, epidemiological evidence, or expert review. Clinical and public health genomics require careful distinction between sequence findings, diagnostic inference, and actionability [65]. A relevant non-LLM example is the public discussion surrounding the New York subway metagenomics study, in which pathogen-related sequence interpretations were later clarified through additional expert analysis and public health review [66,67]. This example illustrates how provisional genomic or metagenomic findings can be misinterpreted when sequence-level evidence is communicated without sufficient validation. In LLM-assisted settings, a similar risk could arise if provisional pathogen-related outputs are presented as confirmed public-health findings before laboratory, epidemiological, and expert review.

Documented failures in LLM-assisted scientific writing, including fabricated or mismatched references, provide a useful contextual warning for clinical and public-health settings [6,13]. These examples are not microbial-genomics benchmarks, but they show that fluent generated text can contain unsupported evidence links. In pathogen genomics, the analogous risk is that a model may convert a provisional sequence finding into an apparent diagnosis, treatment implication, outbreak signal, or infection-control recommendation without laboratory, epidemiological, and expert confirmation.

Accordingly, LLM-assisted outputs should not replace validated AMR prediction tools, diagnostic pipelines, phenotypic susceptibility testing, infection-control procedures, outbreak investigation protocols, or expert microbiological interpretation. Any statement with possible clinical or public-health implications should be treated as provisional until it has been checked against accepted laboratory standards, validated analytical outputs, relevant metadata, and qualified clinical, microbiological, bioinformatic, or public-health expertise.

## 5. Future Directions for Reliable LLM Use in Microbial Genomics

Responsible use of LLMs in microbial genomics requires a shift from informal experimentation toward structured evaluation, transparent reporting, and domain-specific validation [8,68]. A practical path forward is to develop shared benchmark tasks, reporting templates, curated validation datasets, and expert-adjudicated error taxonomies rather than assuming that general LLM performance transfers to microbial-genomics interpretation.

### 5.1. Domain-Specific Benchmarking and Evaluation

A major priority is the development of benchmarks designed specifically for microbial genomics [8]. General LLM benchmarks are unlikely to be sufficient because they often do not test microbial-genomics-specific reasoning, such as gene-to-phenotype interpretation, database-aware AMR explanation, strain-level virulence reasoning, compositional microbiome interpretation, workflow validation, or citation fidelity [4,8].

Future benchmarks should reflect realistic microbial genomics tasks, including genome annotation explanation, AMR interpretation, virulence reasoning, microbiome interpretation, pathway analysis, code generation, workflow debugging, and literature synthesis. Each task should include expert-curated reference answers, predefined scoring criteria, documented input data, database versions, prompt templates, model settings, and evaluation dates [8]. Outcome measures should include factual accuracy, citation precision, hallucination rate, uncertainty handling, code executability, workflow validity, reproducibility, and expert-rated safety [8,13].

Benchmark development should also consider task risk. Low-risk educational explanations may be evaluated differently from high-risk AMR, virulence, or public health interpretation. For example, a coding task may require execution testing and dependency review, whereas a literature synthesis task may require citation checking and claim-level source verification. This risk-sensitive approach would allow future evaluations to assess whether an output is reliable enough for the intended microbial genomics use case.

A proposed empirical validation roadmap for MicrobeGuardGPT should include representative tasks across genome annotation explanation, AMR interpretation, virulence reasoning, microbiome and metagenomics interpretation, literature synthesis, and code or workflow assistance. Candidate inputs could include curated annotation tables, AMR gene outputs from validated tools, virulence-factor reports, mock-community or benchmark metagenomic profiles [69,70], published workflow errors, and literature abstracts with known citation targets. Each task should include a reference answer, expected evidence sources, database versions, acceptable uncertainty language, and a predefined risk category.

Evaluation metrics should be matched to the task. Where reference answers exist, accuracy, precision, recall, F1 score, or Matthews correlation coefficient may be appropriate. For narrative interpretation, expert-rated factual accuracy, biological plausibility, citation validity, source traceability, uncertainty handling, and unsafe-actionable-output rate may be more informative. Coding and workflow tasks should include executability, dependency recording, version capture, output validity, and reproducibility across repeated runs. Expert review should involve at least two domain reviewers when possible, with disagreement resolved by consensus or adjudication and inter-rater agreement reported for benchmark studies.

Error categories should be recorded explicitly, including fabricated references, incorrect gene or taxon names, database-version mismatch, gene-presence-to-phenotype overclaiming, virulence-to-pathogenicity overclaiming, microbiome causality overclaiming, missing uncertainty language, privacy leakage, incomplete provenance, and unrunnable or analytically invalid code. This roadmap would allow future work to evaluate MicrobeGuardGPT empirically while preserving the present manuscript’s scope as a narrative review and conceptual framework.

### 5.2. Curated Database Integration and Retrieval-Augmented Systems

Another important direction is the integration of LLMs with curated microbial genomics databases and validated knowledge resources. Retrieval-augmented systems could require models to consult selected databases, primary publications, software documentation, or validated local documents before generating an answer [16]. In microbial genomics, this approach may help connect LLM outputs to AMR databases, virulence resources, genome annotation systems, taxonomic frameworks, microbiome workflows, and software documentation.

Retrieval-augmented generation can improve evidence access, but it should not be viewed as a complete solution because retrieval quality, citation accuracy, and interpretation remain potential failure points [16]. Future systems should therefore combine retrieval with evidence ranking, source transparency, contradiction checking, database versioning, and expert validation.

Database-linked LLM systems should also record the provenance of retrieved information. In microbial genomics, interpretation can change depending on database release, software version, sequence quality, taxonomic framework, thresholds, and metadata [17]. Future retrieval-augmented systems should therefore document which resources were consulted, which versions were used, and how retrieved evidence supports the final interpretation.

### 5.3. Transparent Reporting of LLM-Assisted Workflows

As LLMs become more common in bioinformatics, authors should transparently report how they were used and how outputs were verified [68]. This is especially important when LLMs contribute to code generation, workflow design, literature synthesis, result interpretation, or manuscript drafting. Reporting should describe how the LLM was used, what information was provided, how outputs were verified, and whether generated material was accepted, revised, or rejected [71].

Existing AI reporting frameworks provide useful models, although microbial genomics will require field-specific adaptation to capture database versions, workflow records, citation verification, code testing, and expert review [68,72].

**Figure 2:**
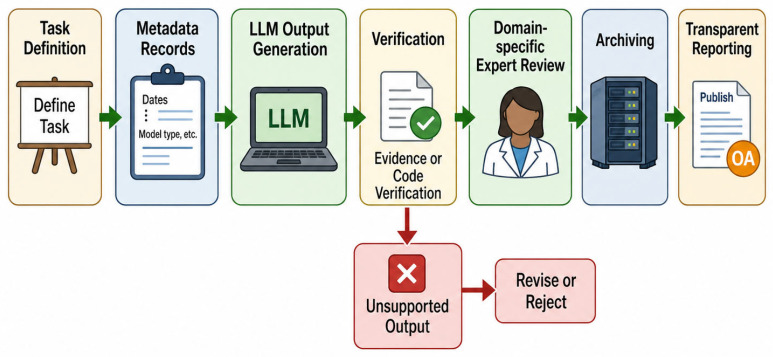
Transparent reporting and validation workflow for LLM-assisted microbial genomics. The workflow outlines task definition, LLM metadata recording, output generation, verification, expert review, and reporting. Unsupported outputs should be revised or rejected before scientific interpretation.

Figure 2 summarizes a practical reporting and validation workflow for LLM-assisted microbial genomics, from task definition and metadata recording to verification, expert review, archiving, and reporting. Unsupported outputs should be revised or rejected before scientific interpretation.

### 5.4. Minimum Reporting Checklist for LLM-Assisted Microbial Genomics

To support reproducibility, review, and auditability, Table 2 proposes minimum reporting items for LLM-assisted microbial genomics workflows, including model identity, purpose of use, input material, prompt records, output handling, citation verification, code testing, database verification, expert review, and availability of validation records.

### 5.5. Human-in-the-Loop Validation, Training, and Governance

Future LLM use in microbial genomics should remain human-in-the-loop [71]. Different tasks require different forms of expertise: bioinformaticians can assess pipeline logic and workflow reproducibility, microbiologists can evaluate taxonomy and biological plausibility, AMR specialists can assess genotype-to-phenotype claims, and metagenomics researchers can review compositional, ecological, and statistical interpretation.

Human validation is particularly important for high-risk outputs. AMR interpretation, virulence reasoning, pathogen surveillance, outbreak-related inference, and public health interpretation may require multidisciplinary review rather than assessment by a single evaluator [4,65]. In these cases, LLM outputs should be treated as provisional until checked against validated pipelines, curated databases, relevant metadata, primary literature, and expert judgment.

LLMs may also support bioinformatics education by helping students understand command-line tools, interpret example outputs, learn programming syntax, and connect computational results to biological meaning. However, training should emphasize critical use, including output verification, hallucination detection, code testing, reference checking, and recognition of biological overinterpretation [13,59]. Governance should therefore combine human review, training, documentation, and clear responsibility for final scientific claims. Unusual, high-risk, or discrepant LLM-assisted outputs should be resolved through expert curation rather than accepted automatically. These priorities provide the basis for MicrobeGuardGPT, the conceptual reliability framework introduced in the next section.

### 5.6. Privacy, Regulatory Frameworks, and Institutional Governance

Governance is especially important when LLM-assisted outputs involve clinical genomics, pathogen surveillance, human-associated microbiome data, or institutional research records. Sensitive genomic, clinical, epidemiological, and metadata inputs should not be uploaded to external systems without appropriate authorization, data-use review, de-identification, security safeguards, and institutional policy alignment. Even when data are de-identified, microbial genomics projects may include location, outbreak, host, or health metadata that require careful access control and audit trails.

International governance frameworks provide useful high-level principles, although they do not replace local legal, ethical, or institutional requirements. The OECD AI Principles [73] emphasize human-centered, trustworthy AI, including human rights, privacy, transparency, robustness, safety, and accountability. ISO/IEC 23894:2023 [74] provides guidance for AI-specific risk management, while ISO/IEC 42001:2023 [75] describes an organizational AI management-system approach. The EU Artificial Intelligence Act, Regulation (EU) 2024/1689 [76], provides a risk-based legal framework for AI systems and emphasizes protection of health, safety, and fundamental rights. For microbial genomics, these frameworks support practical requirements such as human oversight, documented validation, source transparency, role-based access, provenance records, and clear responsibility for final scientific claims.

In practice, laboratories and research groups should define which LLM uses are permitted, which data types may be submitted, who reviews high-risk outputs, how prompts and outputs are archived, how model and database versions are recorded, and how errors are reported. Outputs related to AMR, virulence, outbreak investigation, clinical interpretation, or public-health response should require expert review and should not be treated as autonomous decision support.

## 6. MicrobeGuardGPT: A Conceptual Reliability Framework for LLM-Assisted Microbial Genomics

This review introduces MicrobeGuardGPT as a conceptual reliability framework for assessing LLM-assisted outputs in microbial genomics [8,68]. The framework is intended to support structured evaluation before such outputs are used in biological interpretation, workflow development, literature synthesis, or scientific writing.

**Figure 3:**
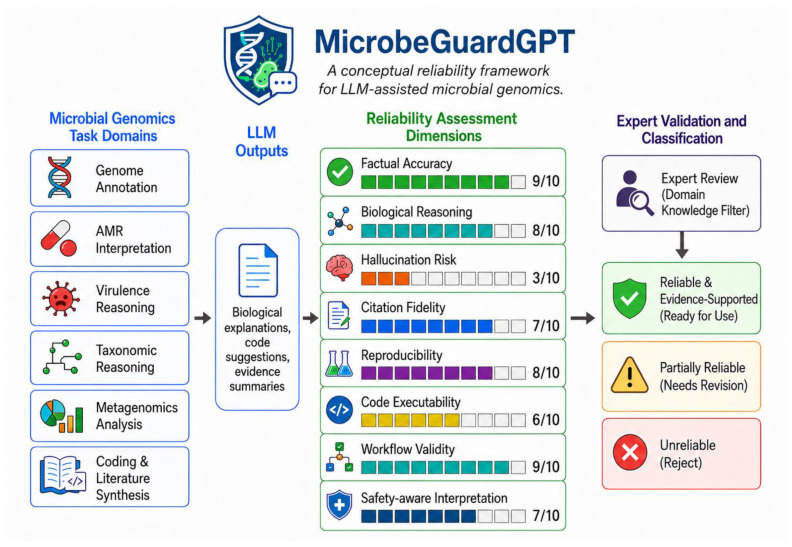
MicrobeGuardGPT: A conceptual reliability framework for LLM-assisted microbial genomics. The framework evaluates LLM-assisted outputs across task domains, evidence quality, biological reasoning, reproducibility, workflow validity, and safety-aware interpretation. Expert review and reliability classification are required before scientific or public health use.

The central principle of MicrobeGuardGPT is that LLM outputs should not be judged only by fluency, readability, or apparent usefulness. In microbial genomics, an acceptable output must also be biologically accurate, evidence-supported, reproducible, transparent, and safe for its intended use [4,17]. As summarized in Figure 3, the framework begins with microbial genomics task domains, proceeds through LLM output assessment across defined reliability dimensions, and ends with expert validation and reliability classification.

### 6.1. Task Domains

MicrobeGuardGPT organizes LLM-assisted microbial genomics tasks into seven broad domains: genome annotation interpretation, AMR interpretation, virulence and pathogenicity reasoning, taxonomic and strain-level reasoning, microbiome and metagenomics interpretation, bioinformatics coding and workflow support, and literature synthesis or scientific writing. These domains differ in risk level, evidence requirements, and validation needs. For example, language refinement or educational explanation may require basic expert checking, whereas AMR, virulence, pathogen surveillance, or public health interpretation requires stronger database verification, contextual review, and domain expertise.

### 6.2. Evaluation Dimensions

MicrobeGuardGPT evaluates LLM-assisted outputs across factual accuracy, biological reasoning, hallucination risk, citation fidelity, reproducibility, code executability, workflow validity, and safety-aware interpretation. These dimensions assess whether an output is not only fluent but also evidence-supported, reproducible, biologically plausible, executable where relevant, and appropriate for its intended use.

For practical use, these dimensions can be linked to an ordinal reliability classification: reliable, partially reliable, unsupported, or unsafe. Table 3 presents a proposed MicrobeGuardGPT rubric for classifying LLM-assisted microbial genomics outputs across these levels.

### 6.3. Worked Example: AMR Interpretation and Reliability Classification

A worked example illustrates how MicrobeGuardGPT could be applied without presenting the framework as empirically validated. Consider a draft LLM-assisted interpretation of a bacterial genome report stating: “The isolate contains blaCTX-M and is therefore resistant to all beta-lactam antibiotics and should be treated as an outbreak threat.” This output is fluent but scientifically unsafe because it converts gene detection into broad phenotype, treatment, and public-health conclusions without sufficient evidence.

The framework would first classify the task domain as AMR interpretation with possible clinical or public-health relevance. The potential risks include genotype-to-phenotype overclaiming, incorrect drug-class scope, missing organism and allele context, absent database versioning, lack of phenotypic susceptibility data, and unsupported outbreak inference. Required validation checks would include confirming the gene call and allele using documented AMR tools or databases such as CARD [32], ResFinder [31], AMRFinderPlus [33], or MEGARes [34]; recording software and database versions; reviewing assembly quality and gene completeness; checking plasmid or mobile-element context where relevant; and comparing with susceptibility testing or validated genotype-to-phenotype rules when available.

A human expert would then revise the interpretation to a cautious evidence-linked statement, for example: “The genome contains a beta-lactamase determinant consistent with possible extended-spectrum beta-lactam resistance, but phenotypic resistance, treatment relevance, and outbreak significance require organism-specific interpretation, validated pipeline output, susceptibility testing where available, epidemiological context, and expert review.” Under MicrobeGuardGPT, the original output would be classified as unsafe because it implies treatment and outbreak actionability without evidence, whereas the revised output would be partially reliable or reliable depending on whether the supporting database, tool, allele, genome-quality, and phenotypic or epidemiological evidence were documented. This example demonstrates the rubric’s intended use as a structured review aid rather than a validated automated scoring system.

### 6.4. Reliability Classification and Expert Validation

Reliability categories should be assigned according to the task, evidence base, and intended use, not according to wording quality alone. In future benchmark studies, classification should ideally be performed by more than one domain reviewer, with disagreements resolved by consensus or adjudication [8]. Where feasible, inter-rater agreement should be reported to assess whether the classification scheme is interpretable and reproducible [72].

Human expert validation is central to this process. Genome annotation and pathway interpretation may require microbiology and functional genomics expertise; AMR interpretation may require microbiology, infectious disease, clinical laboratory, or public health expertise; and microbiome interpretation may require statistical and ecological expertise. Transparent AI reporting and prediction-model assessment guidance also emphasize external evaluation, risk-of-bias assessment, and clear reporting [71,72]. Future implementation of MicrobeGuardGPT could move LLM use in microbial genomics from informal assistance toward structured, auditable, and evidence-aware practice. The framework does not aim to replace expert interpretation but to make the evaluation of LLM-assisted outputs more explicit, reproducible, and accountable.

### 6.5. Limitations of MicrobeGuardGPT

MicrobeGuardGPT remains a conceptual framework and should not be interpreted as a validated benchmark, scoring instrument, or software implementation. It does not yet establish numerical thresholds, validated scoring rules, minimum acceptable performance levels, or formal certification criteria for LLM-assisted microbial genomics outputs. Therefore, it cannot determine whether a specific LLM, prompt, workflow, or interpretation is definitively reliable.

This review does not introduce executable software, a computational pipeline, prompt set, benchmark dataset, or validated scoring instrument. The worked example is illustrative and is provided to show how the proposed rubric could be applied to an LLM-assisted output. Future studies would need to provide full prompts, model and access dates, input files, database and software versions, scoring rules, expert-review procedures, and reproducible benchmark datasets before MicrobeGuardGPT could be evaluated as an empirical tool.

Several limitations should be considered. First, the proposed evaluation dimensions may vary in importance depending on the task. Citation fidelity may be central to literature synthesis, whereas code executability and workflow validity may be more important for bioinformatics pipeline support. Second, reliability classification may be influenced by reviewer expertise, task complexity, data quality, and the availability of validated reference answers. Third, high-risk outputs involving AMR, virulence, pathogen surveillance, or public health interpretation may require multidisciplinary assessment.

The framework also does not automatically solve the technical and practical challenges associated with LLM use. It does not independently prevent hallucination, verify citations, execute code, check database versions, or confirm biological interpretation. These functions would require additional implementation through benchmark datasets, retrieval systems, software tools, code-testing environments, and expert-review workflows. Until such validation is completed, MicrobeGuardGPT should be used as a guide for responsible evaluation, not as a standalone authority for judging LLM reliability.

## 7. Conclusions

LLMs are likely to become increasingly useful in microbial genomics and bioinformatics, particularly for explanation, coding support, workflow assistance, literature synthesis, and scientific communication [1]. However, their outputs cannot be accepted on fluency alone. In microbial genomics, reliable interpretation depends on biological context, validated databases, reproducible workflows, expert review, and careful distinction between prediction, association, phenotype, and clinical relevance.

This review introduced MicrobeGuardGPT as a conceptual framework for evaluating the reliability of LLM-assisted microbial genomics outputs. The framework should be viewed as a proposed structure for risk-aware review, not as a validated benchmark, certification system, or autonomous decision-support tool. Moving forward, responsible use of LLMs in this field will require domain-specific benchmarks, curated database integration, transparent reporting, human-in-the-loop validation, institutional governance, and critical training. Ultimately, the value of LLMs in microbial genomics will depend not on how convincingly they generate scientific language, but on how transparently, reproducibly, and safely their outputs can be verified.

## Figures and Tables

**Table 1:** Risk-stratified use cases for LLMs in microbial genomics and bioinformatics. Risk categories are qualitative and reflect potential consequences of errors, biological-inference depth, database/provenance dependence, reproducibility burden, expert-review requirements, and risk of overinterpretation or hallucination.

Use Case	Example Task	Risk Level	Acceptable LLM Role	Required Safeguard	Reference
Educational explanation	Explaining AMR genes, metagenomic diversity, genome annotation terms, or workflow concepts	Low	Concept explanation and teaching support	Check against textbooks, software documentation, curated databases, or expert notes	[1]
Manuscript language refinement	Improving clarity of methods, results, discussion text, or figure legends	Low to moderate	Editing, restructuring, and readability improvement	Author review and claim-level citation checking	[6]
Literature synthesis	Summarizing studies on AMR, virulence, LLMs, microbiome associations, or bioinformatics workflows	Moderate	Thematic organization and preliminary synthesis	Primary paper verification, DOI/PMID checking, and citation audit	[64]
Code generation	Drafting R, Python, Bash, Perl, Snakemake, or Nextflow scripts	Moderate	Initial script drafting, debugging support, and documentation	Test execution, version control, dependency recording, and workflow review	[57]
Genome annotation interpretation	Explaining predicted gene functions, pathway terms, or annotation outputs	Moderate	Preliminary explanation and report drafting	Curated database checking, sequence evidence, organism context, and genome quality review	[3]
Microbiome and metagenomics interpretation	Explaining taxonomic profiles, diversity results, pathway outputs, or differential abundance findings	Moderate to high	Summary support and cautious interpretation drafting	Check statistical model, metadata, compositionality, database version, and study design	[47]
AMR interpretation	Explaining resistance genes, resistance mechanisms, or genotype-to-phenotype implications	High	Cautious explanation after validated pipeline output	AMR database verification, organism context, susceptibility data where available, and expert review	[4]
Virulence and pathogenicity reasoning	Interpreting virulence-associated genes, pathogenicity islands, secretion systems, or pathogen risk	High	Contextual explanation only	Avoid pathogenicity claims without experimental, genomic-context, or epidemiological evidence	[5]
Clinical or public health inference	Suggesting treatment relevance, outbreak risk, infection-control action, or public health response	Very high	Not suitable as independent decision support	Qualified clinical, laboratory, microbiology, or public health review required	[65]

**Table 2:** Minimum reporting checklist for LLM-assisted microbial genomics workflows.

Reporting Domain	Minimum Item to Report	Example Wording or Record	Purpose	Reference
LLM identity	Model name, provider, version, and date of use	“ChatGPT, provider OpenAI, accessed on [date]”	Supports reproducibility and auditability	[71]
Purpose of use	Whether the LLM was used for writing, coding, troubleshooting, interpretation, or literature synthesis	“Used for language editing and initial code drafting, not for final biological interpretation”	Clarifies how LLM assistance influenced the work	[68]
Input material	Data, text, code, tables, figures, or documents provided to the model	“Genome annotation table and AMR output summary were provided to the model”	Documents the context used to generate outputs	[71]
Prompt record	Key prompts, prompt categories, or prompt logs	“Prompts were archived in Supplementary File X”	Allows later review of generated outputs	[8]
Output handling	Whether outputs were accepted, edited, rejected, or verified	“All LLM-generated claims were manually revised by the authors”	Clarifies author responsibility	[71]
Citation verification	Method used to verify references, DOIs, PMIDs, and claim-level support	“All references were checked using PubMed and journal websites”	Reduces fabricated, mismatched, or unsupported citations	[13]
Code verification	Whether generated code was executed, tested, and version-controlled	“Generated R scripts were tested on example and full datasets”	Supports computational reproducibility	[59]
Database verification	Databases, tools, thresholds, and versions used for biological checking	“CARD and AMRFinderPlus outputs were checked against documented database versions”	Ensures biological interpretation is linked to validated resources	[17]
Expert review	Expertise used to review the biological or computational interpretation	“AMR interpretation was reviewed by a microbiologist or bioinformatician”	Ensures high-risk outputs are not accepted without domain review	[71]
Availability	Whether prompts, outputs, tested code, database versions, and validation notes are shared	“Prompt logs and tested scripts are available in Supplementary File X or repository Y”	Supports transparency, reproducibility, and independent review	[7]

**Table 3:** MicrobeGuardGPT rubric for classifying LLM-assisted microbial genomics outputs.

Dimension	Reliable	Partially Reliable	Unsupported	Unsafe	Reference
Factual accuracy	Biological and technical claims are correct and verifiable	Minor inaccuracies are present, but the main interpretation remains usable	Key claims lack support or cannot be verified	Incorrect claims could seriously mislead interpretation	[8]
Biological reasoning	Interpretation is cautious, context-aware, and biologically plausible	Some context is missing, but there is no major overclaiming	Reasoning is weak, generic, or insufficiently linked to the data	Infers phenotype, pathogenicity, causation, or clinical relevance without evidence	[4]
Hallucination risk	No invented genes, tools, mechanisms, pathways, or references are detected	Minor unsupported wording is present but easily correctable	Important unsupported biological or technical statements are present	Fabricated mechanisms, tools, genes, or references could misdirect analysis	[6]
Citation fidelity	Sources are real, relevant, and support the claims made	Sources are real but only partly support the claims	Sources are weakly relevant, mismatched, or insufficient	Citations are fabricated or seriously misleading	[13]
Reproducibility	Prompt, model, date, output, verification steps, and relevant versions are documented	Partial documentation is available	Poor documentation limits auditability	Output cannot be traced, checked, or reproduced	[17]
Code executability	Code runs correctly in the intended environment and produces expected outputs	Code runs after minor correction	Code fails, lacks dependencies, or is incomplete	Code runs but produces misleading, invalid, or unsafe outputs	[57]
Workflow validity	Workflow follows appropriate bioinformatics logic, including QC, metadata handling, and versioning	Workflow is usable but incomplete	Important QC, metadata, database, or parameter steps are missing	Workflow is analytically invalid or could lead to false biological interpretation	[77]
Safety-aware interpretation	Avoids unsupported clinical, diagnostic, public health, or treatment claims	Minor caution or uncertainty language is needed	Insufficient uncertainty language or expert-review requirement	Suggests actionability without validated evidence or expert review	[71]

## Data Availability

Data sharing is not applicable to this article as no datasets were generated or analyzed during the current study.
